# Increased Zinc Serum Level: New Clues in Babol Stroke Patients, Northern Iran

**DOI:** 10.1155/2018/7681682

**Published:** 2018-02-27

**Authors:** Alijan Ahmadi Ahangar, Payam Saadat, Sona Niroomand, Shayan Alijanpour, Reza Sohrabnezhad, Alireza Firozejahi, Mohamad Ali Biani, Fatemeh Arab, Hamed Hosseinzadeh, Sekine Faraji, Jalal Niroomand

**Affiliations:** ^1^Mobility Impairment Research Center, Babol University of Medical Sciences, Babol, Iran; ^2^Department of Chemistry, Payame Noor University, Tehran, Iran; ^3^Student Research Committee, Isfahan University of Medical Science, Isfahan, Iran; ^4^Ayatollah Rouhani Hospital, Babol University of Medical Sciences, Babol, Iran; ^5^Babol University of Medical Sciences, Babol, Iran

## Abstract

**Background:**

Stroke is the second leading cause of death worldwide. The role of zinc as a new predictor of stroke was considered.

**Methods:**

This prospective study was conducted in Ayatollah Rouhani Hospital within a year on 100 stroke and 100 control patients.

**Findings:**

The difference in zinc serum level in two groups was significant (deficiency: 3 (3%) in patients versus 20 (20%) in control group, normal: 25 (25%) versus 54 (54%), and increased level: 72 (72%) versus 26 (26%); *p* < 0.001). Difference in zinc serum levels was statistically significant with ischemic heart disease (deficiency: 0 cases (0%), normal: 8 cases (24%), increased level: 24 cases (75%), *p* = 0.003). Increases in zinc serum level were significantly correlated with the frequency of hemorrhagic and ischemic patients (deficiency: 3 (3.3%) hemorrhagic versus 0 (0%) ischemic; normal: 19 (21%) versus 6 (60%), increased level: 68 (75.6%) versus 4 (40%); *p* = 0.025). Regression logistics showed that ischemic heart disease (*p* < 0.001; OR = 28.29, %95 CI: 5.53; 144.87), hyperlipidemia (*p* < 0.001; OR = 0.26, %95 CI: 0.12; 0.56), and zinc serum level (*p* < 0.001, OR = 15.53, %95 CI: 4.03; 59.83) each had a significant role.

**Conclusions:**

Babol stroke patients are prone to increased zinc serum level as a new parameter. Ischemic heart disease, increased levels of zinc, and hyperlipidemia were found to be probable predictor factors for stroke in Babol.

## 1. Introduction

Stroke is the second leading cause of death worldwide and imposes enormous financial costs on society [1]. Countries of low and middle income have the largest burden of stroke, accounting for more than 85% of stroke mortalities worldwide [2]. Two-thirds of all strokes occur in developing countries despite their preventable nature. Studies conducted in Iran reported that the incidence of a stroke is about 43–50 patients per 100,000 individuals in the population [3, 4]. Major risk factors for stroke include high blood pressure, diabetes mellitus, ischemic heart diseases, hypercholesterolemia, and smoking [1]. Studies showed that some trace elements necessary due to their role in maintaining the metabolism of neurons can be considered in the diagnosis and treatment of strokes. The imbalance of these trace elements is correlated with increased risk of a stroke [5]. Zinc is an important trace element in biological systems [6]. In recent years the role of zinc as a contributing factor in the pathogenesis of stroke has been considered and it has been commented that some patients with acute cerebral infarction have a reduction in serum levels of zinc [7]. Some research suggests that although zinc is not the initiator of injury, changes in its levels can lead to damage process [8]. Other studies have suggested strokes can cause the release of excess zinc from neurons, such as in disorders that occur in neurotoxicity [9, 10]. Many studies have shown that zinc is an independent risk factor for ischemic strokes [11]; however both neurotoxic and neuron-protective potential of zinc have been reported [5]. Determination of the exact role of zinc in strokes may be able to help in diagnosis, prevention, and treatment of strokes, reducing mortality rates and the costs associated with the disease. Due to contradictory reports about the association of serum zinc level in cases of stroke, this following study was undergone. In this paper, a relationship between zinc serum levels and strokes and its association with the risk factors of stroke was analyzed to explore whether zinc serum levels can be used as a new predictor in stroke patients.

## 2. Materials and Methods

This prospective cross-sectional study was conducted in Ayatollah Rouhani Hospital over the course of a year. In this study, 100 patients (53 females, 47 males) who had had a stroke were enrolled. Patients referred to the hospital with a diagnosis of stroke for one year. The control group was selected from the Amirkola Health and Ageing Project (AHAP, number 892917) matched based on age and sex. Diagnosis of ischemic stroke and its variants is based on common standards in epidemiological studies done on this group of diseases [12]. Stroke was diagnosed by a neurologist, based on the patient's history, neurological examination, and neuroimaging studies which were performed for all stroke patients. Although most stroke cases were ischemic types, Intracerebral Hemorrhage (ICH) was defined as a stroke for which CT Or MRI demonstrated blood within the brain parenchyma, with or without extension into the ventricles or subarachnoid space. Subarachnoid Hemorrhage (SAH) was defined as an abrupt onset of headache, loss of consciousness, or both, with or without focal neurologic signs. In addition, CT scans demonstrated subarachnoid blood. Lumbar punctures were performed in CT-negative patients with an appropriate history. The control group was comprised of 100 (55 females, 45 males) healthy volunteers. The physical examination of control subjects was normal. They were nonsmokers with unremarkable medical histories. The study was approved by the ethics committee of Babol University of Medical Science (BUMS). Informed consent was obtained from each participant or the next of kin before any interview or neurologic examination was conducted. Regarding the sample size formula and its incidence in the region will be approximately 120 cases [4]. Prospective data was collected according to a checklist that included age, gender, onset time of stroke, admission to hospital, vascular risk factors and prior diseases, previous medication, results from laboratory findings, infarct localization, acute-stroke therapy, complications, and medication for secondary prevention. Different intensities were determined based on the Stroke Scale created by the NIH (National Institute of Health) which was defined mild as 1–4, moderate as 5–15, moderate to severe as 16–20, and extreme severe as 21–42. For both the patients and the control group, demographic characteristics, history of ischemic heart disease, hypertension, diabetes mellitus, high blood lipids, smoking, and the zinc serum levels were recorded in a checklist. Subtypes of stroke and their severity upon admission were listed for the patients group.

### 2.1. Exclusion Criteria

Exclusion criteria for stroke cases were hemiparesis or any focal neurological findings due to head trauma, metabolic encephalopathy, and brain mass lesions such as brain tumor or brain abscess, hemiplegic migraine attack, or postictal of seizures [13]. Transient Ischemic Attacks (TIA) cases were excluded from the study. In addition, patients with renal failure, liver failure, and patients taking corticosteroid drugs were excluded.

### 2.2. Blood Samples

Blood samples were collected from all the participants before treatment and placed into empty tubes and immediately stored at 4°C. The zinc serum level was determined by the Randox company kit and Hitachi 912 autoanalyzer Biochemistry at the Ayatollah Rouhani hospital laboratory. The colorimetric method had been used for the determination of zinc levels in 560 nm. Serum level of zinc was defined as 70–120 *μ*g/dl and lower than 70 *μ*g/dl was considered a zinc deficiency while levels above 120 *μ*g/dl were considered increased zinc level [14].

### 2.3. Descriptive Statistical Analysis

The study data were analyzed by applying SPSS for Windows (version 21), according to which the results were expressed as mean value ± standard deviation and parametric variables were compared using an independent sample *T*-test, 2-independent sample chi-square. The results were considered to be statistically significant when the *p* value was less than 0.05.

## 3. Results

Of the 100 stroke patients and 100 control groups, the distribution of sex was 55% females in the control group versus 53% females in stroke patients. The mean age in stroke patients was 70.12 ± 9 versus 70.18 ± 8.5 in control group. The difference in zinc serum level in patients and control group was significant (deficiency, 3 (3%) in patients versus 20 (20%) in control group), normal, 25 (25%) versus 54 (54%), and increased level, 72 (72%) versus 26 (26%), *p* < 0.001) (Table 1). Differences in zinc serum levels were significantly correlated with IHD as an underlying disease (deficiency: 0 cases (0%), normal: 8 cases (24%), and increased level: 24 cases (75%), *p* = 0.003). Increases in zinc serum levels were significantly correlated with increased frequency of hemorrhagic and ischemic patients (deficiency: 3 (3.3%) hemorrhagic versus 0 (0%) ischemic), normal: 19 (21%) versus 6 (60%), increased level: 68 (75.6%) versus 4 (40%), *p* = 0.025). The HLP difference was significant in both the patient and control groups with 50 (50%) in control group versus 35 (35%) in patients, *p* = 0.04). Regression logistics show that IHD (*p* < 0.001, OR = 28.29, %95 CI: 5.53; 144.87), HLP (*p* < 0.001, OR = 0.26, %95 CI: 0.12; 0.56), and zinc serum level (*p* < 0.001, OR = 15.53, %95 CI: 4.03; 59.83) had a significant role (Table 2). First background disease was HTN, 65 (65) cases in stroke patient versus 57 (57) cases in control group, *p* = 0.31). Differences in the lower and upper of cutoff point of zinc (serum level = 95) were not significant (*p* > 0.05).

## 4. Discussion

There was a significant difference between the zinc serum levels measured in the patient group and those of the control group. 87% of the control group had a zinc deficiency while 73.5% stroke patients had increased zinc serum levels. In Qi et al. study, opposite direction about role of zinc was concluded that can lead to protective or harmful result [11]. The De Paula et al. study expressed that enormous and transient zinc accumulation during cerebral ischemia was significantly involved in brain damage through promotion of neuronal apoptotic death, so removing zinc can be a means of reducing ischemic brain injury [14]. Tomas-Sanchez et al. found that despite the neuroprotective role of lower doses of zinc against cerebral ischemia, zinc accumulation leads to cytotoxicity, neuroinflammation, neuronal death, and cerebral dysfunction [15]. It also stated that the zinc serum level can be used in the detection and prevention of stroke [5, 7, 16]. Gower-Winter and Levenson showed that low levels of zinc are associated with increased stroke severity and suggested zinc supplements be used to prevent ischemic strokes [17]. The Galasso and Dyck mentioned that zinc was able to act both as a mediator and as a destructive agent in nerve cell function during cerebral ischemia and concluded there were two sides to the function of zinc [18]. In the Kitamura et al. study, a low concentration of zinc was found to lead to the inhibition of calcium influx through glutamate receptor and caused cell death. On the other end of the spectrum, high concentrations of zinc decreased the viability of nerve cells [19]. Prakash et al. concluded that a normal level of zinc was suitable for health but that a zinc homeostasis disorder can lead to nerve cell death pathways [20]. So the dual effect of zinc might be due to concentration dependence and different cutoff points used across various studies mean that the authors are unable to compare conclusions accurately. In this study just 3% of patients with stroke had a zinc deficiency while this rate in the Bhatt studies was 35.7% [21]. This shows that Babol stroke patients are prone to increased zinc serum levels rather than a deficiency. According to analysis of multivariate logistic regression in this study, IHD, zinc increased levels and HLP were found to be probable prediction factors for stroke in Babol stroke patients as 30% of stroke patients had IHD and 77% stroke patients had increased zinc levels. In the Mielcarz et al. study, an increase in zinc concentration was found to correlate with increased risk of IHD [22]. This finding was in line with this study and showed the necessity of control and prevention of this parameter. Differences in zinc serum levels were statistically significantly correlated with IHD, especially as increased levels were seen in 75% of patients. The Gower‐Winter and Levenson study showed that dietary zinc can cause oxidative stress in vascular endothelial dysfunction and can lead to cardiovascular disease [17]. On the other hand, Soinio et al. concluded that low serum levels of zinc can be an independent risk factor of Coronary Artery Disease [23]. It appears that changes in the concentration of zinc, especially increased levels of zinc, can lead to heart disease. The type of stroke showed significant differences correlated with zinc serum levels in that 75% of ischemic stroke patients had increased zinc serum which was in contrast with the Karadas et al. study conducted in Turkey. They found that ischemic or hemorrhagic type of stroke does not affect the amount of zinc [16]. Higher zinc serum level in ischemic patients can be due to high prevalence of ischemic rather than hemorrhagic disease. The difference in zinc serum level had statistical significance with HLP and 68.6% of patients with zinc increased levels had HLP. Neggers et al. did not observe any significant correlation between levels of zinc with total serum cholesterol [24]. A contradictory study reported using zinc supplements in patients with hyperlipidemia which was found to increase zinc serum levels and decrease serum lipid profiles. These results recommended zinc supplements as an effective treatment of hyperlipidemia [25]. That was in contrast with this study. Result show direct correlation between increase of zinc and HLP; on the other hand HLP is more often seen in control group than patients. It can be related to demographic characteristics. Prevalence of HTN in stroke patients was 65% in line with Tomas-Sanchez et al. study in India. Perhaps it can be related to food culture or other similar factors in two regions that lead to high systolic and diastolic blood pressure [15]. The Galasso and Dyck study concluded that patients with antihypertension drug consumption had lower zinc serum levels in comparison to patients with no drug consumption for HTN [18]. Due to the high prevalence of HTN and heart disease under drug treatment and because of a lack of information about patient lifestyle, we cannot accurately compare HTN and zinc serum levels to separate their respective roles in strokes. In our study insignificant differences between zinc serum levels and diabetes among stroke patients and the control group were not observed. However, the relationship between the prevalence of CAD and diabetes and low intake of zinc was reported [26].

Generally, on the basis of surveyed studies, several factors may be involved in the effect of zinc level in stroke. This includes the method of study, assessing the level, storage conditions of samples before tests, kits with different sensitivity, and specificity in the age range of the studied population. These issues and the complicated effects of zinc lead to an ambivalence regarding the conclusion. This study had limitations which included a small sample size and lack of documentation with regard to the consumption of zinc supplements by patients.

## 5. Conclusion

Results showed that Babol stroke patients were prone to increased zinc serum level than deficiency which was a new clue to the diverse roles of zinc. Increased level of zinc can be defined as new laboratory parameter in stroke. IHD, increased levels of zinc, and HLP were found to be probably predictor factors for stroke in Babol stroke patients. It seems that serum levels of zinc could be a new target in the investigation for the treatment of stroke. It is suggested that the zinc serum levels should be considered with more sensitivity during hospital admission and that health official should pay more attention to this parameter in population and it can be used in screening and routine lab test in stroke patient.

## Figures and Tables

**Table 1:** Evaluation of different variable with severity and statistical society.

Variable	subgroup	Statistical Society		*p* value	Severity			*p* value
		Control	Patient		Mild	Moderate	Severe	
Sex	Male	45 (45)	47 (47)	0.87	34 (46)	10 (42)	3 (100)	0.16
	Female	55 (55)	53 (53)		39 (54)	14 (58)	0 (0)	

Age	60–69	55 (55)	54 (54)	0.99	37 (51)	16 (67)	1 (33.3)	
	70–79	26 (26)	27 (27)		21 (29)	5 (20)	1 (33.3)	0.64
	Above 80	19 (19)	19 (19)		15 (20)	3 (13)	1 (33.3)	

IHD	No	98 (98)	70 (70)	<0.0001	51 (70)	18 (75)	1 (33.3)	
	Yes	2 (2)	30 (30)		22 (30)	6 (25)	2 (66.7)	0.33

DM	No	68 (68)	62 (62)	0.45	47 (64)	14 (58)	1 (33.3)	
	Yes	32 (32)	38 (38)		26 (36)	10 (42)	2 (66.7)	0.50

HTN	No	43 (43)	35 (35)	0.31	27 (31)	8 (33)	0 (0)	
	Yes	57 (57)	65 (65)		46 (69)	16 (67)	3 (100)	0.41

HLP	No	50 (50)	65 (65)	0.04	47 (64)	17 (71)	1 (33.3)	
	Yes	50 (50)	35 (35)		26 (36)	7 (29)	2 (66.7)	0.42

Smoker	No	82 (82)	72 (72)	0.13	56 (77)	15 (62)	1 (33.3)	
	Yes	18 (18)	28 (28)		17 (23)	9 (38)	2 (66.7)	0.12

Zinc serum level	Under 70	20 (20)	3 (3)	<0.0001	2 (2.7)	1 (4.2)	0 (0)	0.79
	70–120	54 (54)	25 (25)		20 (27.4)	5 (20.8)	0 (0)	
	Above 120	26 (26)	72 (72)		51 (69.9)	18 (75)	3 (100)	

Type	Ischemic	0 (0)	90 (90)	-	67 (91)	21 (87)	2 (66.7)	0.32
	Hemorrhagic	0 (0)	10 (10)		6 (9)	3 (13)	1 (33.3)	

*Note*. Ischemic heart disease (IHD), diabetes mellitus (DM), hypertension (HTN), and hyperlipidemia (HLP).

**Table 2:** Evaluation of zinc serum level with different variables in the two groups.

Variable	Group	Zinc serum level			*p* value
		Microgram/deciliter			
		Increased level	Normal	Deficiency	
IHD	Control	1 (50)	1 (50)	-	**0.003**
	Patient	23 (76.7)	7 (23.3)	-	
Stroke	Ischemic	68 (75.6%)	19 (21%)	3 (3.3%)	
	Hemorrhagic	4 (40%)	6 (60%)	0 (0%)	**0.025**
HLP	Control	17 (34)	24 (48)	9 (18)	**0.04**
	Patient	24 (68.6)	11 (31.4)	-	

Data are odds ratio (99% CI). Models are adjusted for age, sex, diabetes, smoking, HLP, and zinc serum level. Data are odds ratio (99% CI). Models are adjusted for age, sex, Diabetes, smoking, HLP, and zinc serum level. IHD: ischemic heart disease and HLP: hyperlipidemia.

## Additional Points

*Highlights*. Increased level of zinc can be defined as a new laboratory parameter in stroke. Zinc serum level can be an independent risk factor for stroke. Serum levels of zinc could be a new target in the investigation for the treatment of stroke. Zinc serum levels higher than 120 mg/dl can be risk factors for stroke. Difference in serum levels of zinc in stroke patients with IHD and HLP were statistically significant.
